# Indications for the Inlay

**Published:** 1909-07-15

**Authors:** J. F. Nyman


### INDICATIONS FOR THE INLAY.

BY J. F. NYMAN, D.D.S.

There are cavities to which the chiseling away of the frail enamel walls imparts a natural retentive shape, so that very little cutting of any kind is required for the proper retention of a gold filling; but it would require extensive cutting to insert a gold inlay or any other inlay. Under those circumstances I do not believe that we are justified in putting in an inlay. I find many large compound cavities with some peculiar extension of caries. To prepare such cavities for a gold inlay would require so much cutting that it would simply make me look aghast at an operator who would do it; but when I can solve the problem nicely for the patient and myself by cleaning out the caries in the little odd extension, filling it temporarily, and later removing the cement and inserting a small gold filling alongside of my inlay I combine the two.

In those extensive operations that mean hours and hours of inserting gold foil and large fractions of hours of polishing, I believe the modern gold inlay is better for the tooth than the finest gold filling, because the fact that the tooth must be kept so profoundly dry for such a long time exerts a very deleterious effect on both dentin and enamel. In such a tooth an injury occurred which perhaps was not apparent at the time, nor immediately afterward, but it manifested itself some time later, when to the dismay of the operator and to the consternation of the patient a whole section of enamel and dentin broke off. There is a flow to gold foil, and even to gold in combination with platinum. Under the incessant stress of mastication the gold foil spreads, and if the force should be exerted at a certain angle, within a short time the buccal or lingual wall will be broken off.

With the gold inlay we avoid in the first place this excessive dryness. In the second place, we avoid weakening the side walls, and in the third place, we avoid ultimate thermal shock, because we have the interposition of the layer of cement, which acts as an insulation against heat. I place my reliance on such a retention, which by its mechanical nature safeguards the inlay against the stress of mastication, so that it could not be dislodged by the force of mastication even if there were no cement there. I never pretend to depend upon the adhesiveness of the cement. Truly, the adhesiveness of the cement does retain the inlay against the force of gravity as exerted by its own weight or the force that might be developed by suction, but aside from that, I do not believe that cement should ever be relied upon to hold in an inlay.

If you will prepare a cavity correctly, and follow conscientiously all the steps of the cast gold inlay process, you will produce an inlay which can scarcely be distinguished from a filling. I have examined gold inlays made by the hands of the most capable operators under strong hand magnifying glasses, and although these inlays were set with a cement which did not correspond with the gold inlay or the tooth-structure, the operator had so carefully burnished the gold over the margin that he practically covered up the cement line, and produced an inlay that will probably never show any signs of dissolution on the margin.

Many of the old operators decry gold inlays, because they say it is no art to make a wax model of a filling, and reproduce it outside of the mouth. Instead of it being no art at all to make a wax model for a cavity, I think it is the most delicate of any of the steps of the gold inlay method, the most difficult part of the whole gold inlay operation.

We do not employ the gold inlay method because it is an easier operation. Provided the form of a cavity is favorable, it is the most humane method of any that can be developed. It is far easier for many operators to cut away the tooth structure with wheel and bur and disk, than it is to properly condense and finish golf foil, and I am sorry to confess that I have watched many men work feeling that their predominating motive for deciding what they were to do in that cavity was their desire to perform the easiest operation, and their conviction that they could put in an inlay better than they could pack in and finish foil.

As far as porcelain inlays are concerned, it has been my experience that they are no longer to be regarded as a permanent practical repair of the teeth. They are only to be used in those cases in which the esthetic phase is the predominant factor. You cannot construct or set a porcelain inlay that will ever begin to be such a permanent protection to a tooth as a gold inlay is, because of the fact that it is impossible to prepare the margins of the inlay and of the cavity in such a way that their preparation guarantees permanent results. I have come to regard the porcelain inlay as the most prominent of the esthetic measures for repairing teeth that occupy a conspicuous position in the mouth. I feel that in a cavity in which cement would last for a year or two, we may expect a porcelain inlay to be retained for from three to five years, and I am cautioning all my patients now that they must keep a very careful watch on any porcelain inlays in their mouths that have been in for more than two or three years, because in the natural order of things they may drop out. This is my conviction gained by experience and thought on this subject.—June *Cosmos.*

